# Supplementary material for: Delta neutrophil index and shock index can stratify risk for the requirement for massive transfusion in patients with primary postpartum hemorrhage in the emergency department
Source: PLoS One. 2021 Oct 15;16(10):e0258619. doi: 10.1371/journal.pone.0258619 (PMC8519472; doi:10.1371/journal.pone.0258619)
Supplement: S1 Table — (DOCX) [file pone.0258619.s001.docx]

**Supplement**

**Table S1.** Univariate logistic regression analysis for clinical factors associated with the requirement for massive transfusion in patients with primary postpartum hemorrhage

| **Variables** | **Massive transfusion** | |
| --- | --- | --- |
|  | **OR (95% CI)** | ***p*** |
| Age (per 1 year increase) | 1.122 (1.037-1.215) | 0.004* |
| **Parity** |  |  |
| Primipara | Reference |  |
| Multipara | 1.244 (0.697-2.220) | 0.46 |
| **Type of delivery** |  |  |
| Vaginal delivery | Reference |  |
| Cesarean section | 1.930 (1.078-3.456) | 0.027* |
| **Initial mental status** |  |  |
| Alert | Reference |  |
| Non-alert | 24.108 (2.843-204.450) | 0.004* |
| **Initial vital signs** |  |  |
| Shock index (per 1) | 56.292 (17.269-183.498) | <0.001* |
| Systolic blood pressure (per 1 mmHg) | 0.954 (0.938-0.969) | <0.001* |
| Diastolic blood pressure (per 1 mmHg) | 0.948 (0.926-0.970) | <0.001* |
| Heart rate (per 1 bpm) | 1.038 (1.024-1.053) | <0.001* |
| Body temperature (per 1^o^C) | 0.487 (0.305-0.777) | <0.001* |
| **Initial laboratory data** |  |  |
| White blood cell count (per 10×3/μL) | 1.000 (0.958-1.043) | 0.983 |
| Hemoglobin (per 1 g/dL) | 0.759 (0.653-0.881) | <0.001* |
| Platelet count (per 10×3/μL) | 0.986 (0.981-0.991) | <0.001* |
| Albumin (per 1 g/dL) | 0.181 (0.097-0.339) | <0.001* |
| Total CO_2_ (per 1 mmol/L) | 0.802 (0.723-0.888) | <0.001* |
| Delta neutrophil index (per 1) | 1.490 (1.305-1.701) | <0.001* |
| **Amount of blood transfusion** |  |  |
| Packed red blood cells (per 1 unit) | 2.422 (1.874-3.131) | <0.001* |
| Fresh frozen plasma (per 1 unit) | 2.257 (1.849-2.754) | <0.001* |
| Platelet concentrate (per 1 unit) | 1.509 (1.356-1.679) | <0.001* |
| **Clinical outcome** |  |  |
| Embolization | 1.775 (0.886-3.556) | 0.106 |
| Hysterectomy | 65.981 (8.471-513.938) | <0.001* |
| Emergency surgery | 28.380 (12.265-65.672) | <0.001* |
| In-hospital death | 11.140 (0.118-999.999) | 0.299 |

**p*<0.05. SBP, systolic blood pressure; DBP, diastolic blood pressure; WBC, white blood cell; OR, odds ratio; CI, confidence interval
