# Supplementary material for: Delta neutrophil index and shock index can stratify risk for the requirement for massive transfusion in patients with primary postpartum hemorrhage in the emergency department
Source: PLoS One. 2021 Oct 15;16(10):e0258619. doi: 10.1371/journal.pone.0258619 (PMC8519472; doi:10.1371/journal.pone.0258619)

Figure2

Click here to access/download;Figure;PPH-fig 2.jpg

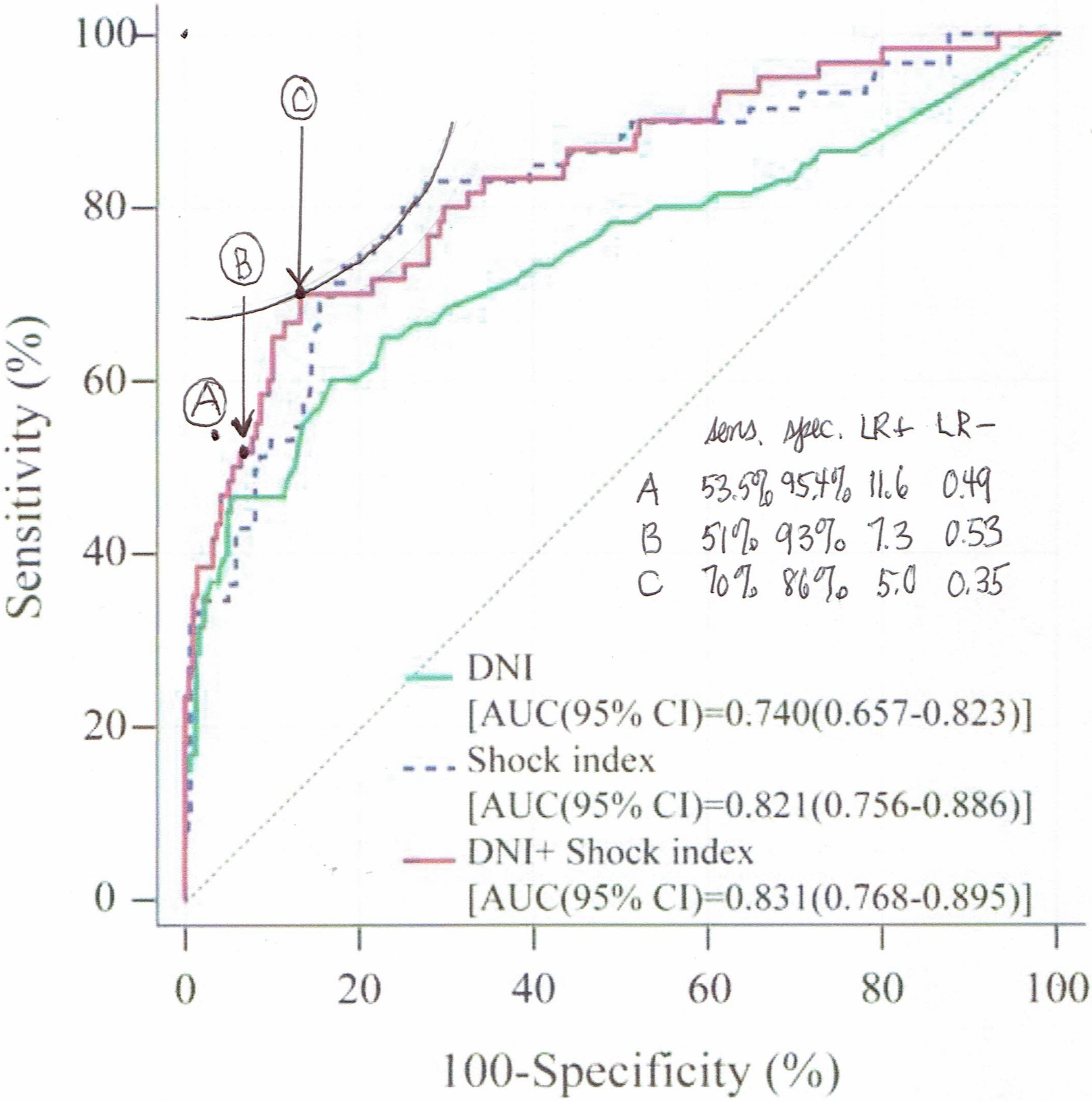

Supplement: Attachment — Submitted filename: ROC - 8.3.2021.pdf [file pone.0258619.s004.pdf]
