# Supplementary material for: Delta neutrophil index and shock index can stratify risk for the requirement for massive transfusion in patients with primary postpartum hemorrhage in the emergency department
Source: PLoS One. 2021 Oct 15;16(10):e0258619. doi: 10.1371/journal.pone.0258619 (PMC8519472; doi:10.1371/journal.pone.0258619)
Supplement: Attachment — Submitted filename: Response to reviewer.docx [file pone.0258619.s005.docx]

***** Response to reviewer’ s comments *****

***Delta neutrophil index and shock index can stratify risk for the requirement for massive transfusion in patients with primary postpartum hemorrhage in the emergency department***

**Response to reviewer #4’s comments**

Reviewer #4: Title: “Delta neutrophil index and shock index can predict the requirement…” I think the use of the word “predict” offers something to the readers that the study does not actually provide. Perhaps better would be, “Delta neutrophil index and shock index can risk stratify the requirement…” Although the information in the “Abstract” is correct, it is of little interest to the clinician. That the odds ratios are statistically significant is of little help to the clinician about to care for the next patient with PPH. It would be better to substitute information about likelihood ratios since they can be used directly by the clinician to take care of the patient. Even if the authors choose to retain odds ratios in the abstract, they should also include the information that values below the cutoff were not helpful, whether they were statistically significant or not.

**Response:** Thank you for your astute comments and valuable suggestions. We agree with the reviewer’s opinion.

First, we have revised the title.

*Delta neutrophil index and shock index can stratify risk for the requirement for massive transfusion in patients with primary postpartum hemorrhage in the emergency department*

Second, we have substituted odds ratios with likelihood ratios.

Results: Multivariable logistic regression revealed that the DNI and SI were independent predictors of MT. The optimal cut-off values of ≥3.3% and ≥1.0 for the DNI and SI, respectively, were significantly associated with an increased risk of MT (DNI: positive likelihood ratio [PLR] 3.54, 95% confidence interval [CI] 2.5–5.1 and negative likelihood ratio [NLR] 0.48, 95% CI 0.4–0.7; SI: PLR 3.21, 95% CI 2.4–4.2 and NLR 0.31, 95% CI 0.19–0.49). The optimal cut-off point for predicted probability was calculated for combining the DNI value and SI value with the equation derived from logistic regression analysis. Compared with DNI or SI alone, the combination of DNI and SI significantly improved the specificity, accuracy, and positive likelihood ratio of the MT risk.

I had previously made the comment about the number of significant figures: “…SBP (94.267…). Reporting SBP to 3 significant figures is not needed. Better to report the results for blood pressures and heart rates as whole numbers. This will also make the paper easier to read.

The response was: “Thank you for your helpful comment. Accordingly, we have revised the systolic blood pressure and heart rate values as whole numbers in the revised manuscript.” However, I did not see where this was actually done. Please follow up with this. Furthermore, Table 1 would be easier to read if values were rounded out to whole numbers. This would not lose anything in terms of conclusions reached by the study. For example, the first entry, 32.70±3.95 is better reported as 33 ± 4. There are other places in the body of the paper where results are given with an inordinately large number of significant figures. This just makes the paper harder to read, does not add anything to the interpretation of the results and is actually not justified from a statistical point-of-view. For example, “…with a specificity of 83.03% (95% CI: 78.05–88.01)” implies that the specificity of 83.04 is incorrect, whereas the 95% CI includes that value. I would be better to say, “…with a specificity of 83% (95% CI: 78-88).” Furthermore, I really don’t think anyone cares whether the specificity is 83.03% or 83%.

**Response:** Thank you for your pertinent comments. We sincerely apologize for our oversight in the previous manuscript version. We have revised the values to whole numbers in this manuscript version.

Regarding the following sentence: “On combining a DNI value of ≥3.3 with an SI value of ≥1.0, the specificity and PLR increased to 95.41% (95% CI; 92.63–98.19) and 11.63 (95% CI: 6.07–22.27), respectively.” I had some difficulty with data taken from the DNI+SI ROC graph, since the point on the graph where sensitivity = 53.5% and specificity = 95.4% is not actually on the line (see graph in attachment, point A). The authors did not address this concern in their response to the initial review even though this is crucial for interpreting the data. Their result (PLR for DNI+SI = 11.63) is much higher than the 7.3 or 5.0 that I calculated using points that are actually on the plotted line (attached graph, points B and C). The authors need to address this since you cannot arbitrarily choose any point on the graph to make the calculations. You need to use a point that is actually on the line in the graph. Please make this correction in the manuscript.

**Response:** Thank you for your remarkable comments and insightful suggestions. As you mentioned, on combining a DNI value of ≥ 3.3 with an SI value of ≥ 1.0, an optimal cut-off point could not be represented using ROC curves. Therefore, we determined arbitrary point as an optimal cut-off point by combining the optimal cut-off points of both DNI value of ≥ 3.3 and SI value of ≥ 1.0. We believe that this optimal cut-off point can help in the clinical decision-making. However, we agree that we should measure sensitivity, specificity, and likelihood ratio based on the optimal cut-off point obtained on combining the DNI value and SI value represented on the ROC curve. We discussed this point with our statisticians and added the optimal cut-off point to the ROC curve using the equation derived from logistic regression.

To stratify risk for the requirement for massive transfusion by combining the DNI value and SI value in patients with primary postpartum hemorrhage in the ED, we used the following equation derived from logistic regression.

Predicted probability = 1/(1+exp (5.348-0.2464*DNI-3.2691.*SI)

Based on this equation, we calculated predicted probability for combining the DNI value and SI value. We determined the optimal cut-off point for predicted probability (DNI+SI) to be ≥ 0.249.

When we stratified risk for the requirement for massive transfusion with the predicted probability (≥ 0.249) by combining the DNI value and SI value in patients with primary postpartum hemorrhage in the ED, the application of the value significantly improved the specificity, sensitivity, and PLR for massive transfusion in comparison with SI alone. In addition, it also improved PLR and NLR in comparison with DNI alone. We have marked a “cross” on the ROC curve of Figure 2. This point represents the optimal cut-off point based on the predicted probability (DNI+SI) of ≥ 0.249.

We have deleted texts pertaining to the optimal cut-off point derived by combining the optimal cut-off points of both DNI value of ≥3.3 and SI value of ≥1.0 from the manuscript according to reviewer’s comment. We have added the optimal cut-off point to the ROC curve using the equation derived from logistic regression to the revised manuscript.

Method

In addition, to stratify risk for the requirement for massive transfusion by combining the DNI value and SI value, we used the equation derived from logistic regression analysis. Based on this equation, we calculated the predicted probability for combining the DNI value and SI value. We determined optimal the cut-off point for the predicted probability. Based on this optimal cut-off point, we measured sensitivity, specificity, PLR, NLR, and accuracy.

Results

The predicted probability derived from the logistic equation was computed for each patient. When the predicted probability of the equation [Predicted probability = 1/(1+exp(5.348-0.2464*DNI-3.2691.*SI)] containing the combination of the DNI and SI was more than 0.249, the specificity was 87% (95% CI: 82–91), sensitivity was 70% (95% CI: 58–82), accuracy was 83% (95% CI: 79–88), PLR was 5.26 (95% CI: 3.6–7.7), and NLR was 0.35 (95% CI: 0.2–0.5) for MT. The combination of the DNI and SI significantly improved the specificity (DNI vs DNI + S, *p*<0.001; SI ≥1.1 vs DNI + SI, p<0.001), accuracy (DNI vs DNI + SI, *p*<0.001; SI ≥1.1 vs DNI + SI, p<0.001), and PLR (DNI vs DNI + S, *p*<0.001; SI ≥1.1 vs DNI + SI, p=0.038; using the GEE method) for the requirement for massive transfusion when compared with either DNI or SI (Table 3).

**Figure 2.** Receiver operating characteristic curves of the delta neutrophil index, shock index, and the combination of the delta neutrophil index and shock index for the requirement for massive transfusion in patients with primary postpartum hemorrhage

DNI, delta neutrophil index; SI, shock index; AUC, area under the curve; CI, confidence interval

**Table 3.** Prognostic value of the shock index, delta neutrophil index, and combined delta neutrophil index with shock index for predicting the requirement for a massive transfusion in patients with primary postpartum hemorrhage.

| **Variable** | **Sensitivity (95% CI)** | **Specificity (95% CI)** | **Accuracy (95% CI)** | **PLR** | **NLR** |
| --- | --- | --- | --- | --- | --- |
|  |  |  |  | **(95% CI)** | **(95% CI)** |
| 1> Delta neutrophil index ≥ 3.3 | 60 | 83 | 78.1 | 3.54 | 0.48 |
|  | (47.6–72.4) | (78.1–88.0) | (73.2–82.9) | (2.47–5.06)) | (0.35–0.66) |
| 2> Shock index ≥ 1 | 76.7 | 76.2 | 76.3 | 3.21 | 0.31 |
|  | (66.0–87.4) | (70.5–81.8) | (71.3–81.3) | (2.44–4.23) | (0.19–0.49) |
| 3> Probability equation* ≥ 0.249 | 70 | 86.7 | 83.1 | 5.26 | 0.35 |
|  | (58.4–81.6) | (82.2–91.2) | (78.7–87.5) | (3.61–7.67) | (0.23–0.51) |
|  | Comparison of P value | | | | |
| 1 vs 3 | 0.038* | <0.001* | <0.001* | <0.001* | 0.853 |
| 2 vs 3 | <0.001* | <0.001* | <0.001* | <0.001* | 0.015* |

*P<0.05. PLR, positive likelihood ratio; NLR, negative likelihood ratio; CI, confidence interval.

§Equation of predicted probability = 1 / (1+exp (5.348-0.2464*DNI-3.2691*SI)
